# Hover Fly Eyes and Antennae Are Sexually Dimorphic but Do Not Tradeoff

**DOI:** 10.17912/micropub.biology.002218

**Published:** 2026-08-20

**Authors:** George J. Todd, Aimee S. Dunlap

**Affiliations:** 1 Biology, University of Missouri–St. Louis, St Louis, MO, United States; 2 Harris World Ecology Center, St Louis, MO, United States

## Abstract

Sensory systems are energetically expensive and may evolve through tradeoffs between modalities. We tested the sensory tradeoff hypothesis in four hover fly species by examining allometry and sexual dimorphism in eye and antennal (funiculus) size. Across species, larger flies exhibited larger sensory structures and males generally possessed relatively larger eyes and funiculi than females after controlling for body size. However, we found no consistent negative relationship between eye and antennal investment within species. These findings suggest that sensory investment in hover flies is shaped more strongly by allometric and sex-specific selection pressures than by compensatory tradeoffs between sensory modalities.

**Figure 1. Allometric relationships and sensory tradeoffs in four hover fly species:** Relationships among body size, sensory structures, and sex in four hover fly species. (A) Log-transformed eye size versus thorax width. (B) Log-transformed funiculus size versus thorax width. (C) Log-transformed eye size versus funiculus size. Points represent individual flies and are colored by species and shaped by sex (circles = females; triangles = males). Regression lines are shown separately for each species and sex (solid = females; dashed = males). **Table 1** . Allometric scaling, sexual dimorphism, and sensory tradeoffs in hover fly sensory structures. Standardized major axis (SMA) slopes and p-values describe relationships between log-transformed sensory traits and thorax width. Sex effects were assessed using linear models controlling for thorax width, with sexual dimorphism indicating the direction of significant differences (M > F = males larger; F > M = females larger). Tradeoff slopes and p-values represent relationships between log-transformed eye and funiculus size while controlling for thorax width and sex. n.s. indicates non-significant results.

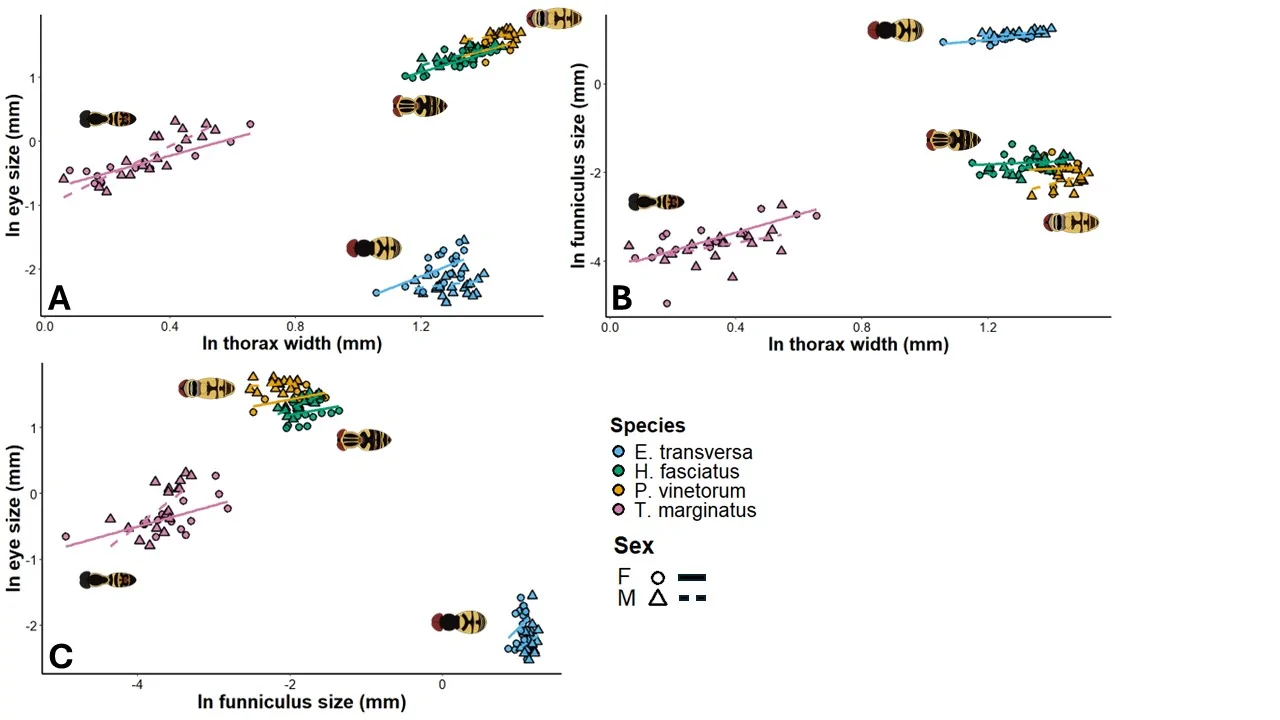



**Table:** 

Species	Trait	SMA slope	SMA p-value	Sex effect p-value	Sexual Dimorphism	Tradeoff slope	Tradeoff p-value
P. vinetorum	Eye	2.77	0.008	< 0.001	M > F	0.207	0.135
	Funiculus	5.34	0.847	0.01	F > M		
E. transversa	Eye	3.85	0.241	< 0.001	F > M	0.281	0.768
	Funiculus	1.18	< 0.001	< 0.001	M > F		
H. fasciatus	Eye	1.83	< 0.001	0.005	M > F	0.125	0.244
	Funiculus	2.21	0.071	0.1	n.s.		
T. marginatus	Eye	2.15	< 0.001	0.266	n.s.	-0.057	0.502
	Funiculus	2.79	< 0.001	0.175	n.s.		

## Description

Sensory systems are beneficial, and indeed critical, but very energetically expensive. These costs and benefits create a dynamic that likely shapes the function, design and evolution of sensory systems as well as behavior. Thus, different sensory structures could increase in size and complexity or shrink through evolutionary time depending on environmental factors creating selection pressures on an animal. The sensory tradeoff hypothesis posits that as one sensory modality increases in resource investment in an animal species, another will decrease in resource investment (e.g. Barton et al. 1995). A classic example in vertebrates is in the Mexican cavefish *Astyanax mexicanus* . Cave-dwelling ecotypes of this species exhibit decreased investment in eye tissue (Moran et al. 2015) while also exhibiting enhanced lateral line mechanosensory systems (Rodríguez‐Morales 2024). Although a disproportionate amount of research investigates this dynamic in vertebrates (Hodos & Butler 1997, Oteiza & Baldwin 2021) there have been some studies in recent years testing this hypothesis in invertebrates (Stöckl et al. 2016, Keesey et al. 2019, Moradinour et al. 2021, Jelley & Barden 2021). One useful context to test the sensory tradeoff hypothesis in invertebrates is within the dynamics of plant-pollinator interactions. Pollination is an important and complex biological process (Real 2012, Ollerton 2017) and involves interpreting a complex array of sensory signals and cues from the pollinator’s perspective (Balamurali et al. 2015). Many pollinators rely heavily on vision (Arnold 2010) and olfaction (Rusch et al. 2016) during pollination. There is evidence for these sensory modalities trading off in numerous species (Keesey et al. 2019, Stöckl et al. 2016, Rozanski et al. 2022, Streinzer & Spaethe 2014). Despite this insight, one key pollinator group has never been tested with the sensory tradeoff hypothesis: hover flies. Hover flies (Syrphidae) are the second most important pollinating group behind bees (Rader et al. 2016) and they readily use both vision and olfaction during pollination (Primante & Dötterl 2010, Hannah et al. 2019). Additionally, we have evidence that hover fly visual systems are unique among Dipteran flies and may more closely resemble visual systems of bees (Hannah et al. 2019). Thus, we tested the sensory tradeoff in several species of hover fly at the sensory organ level (i.e., eyes/antennae). We first hypothesized that hover fly sensory organs scale allometrically with body size. Specifically, we predicted that larger hover flies would exhibit larger eyes and antennae. We also hypothesized that hover flies are sexually dimorphic in their sensory organ size and predicted that female flies would exhibit relatively larger antennae while males would exhibit relatively larger eyes. Finally, we tested the sensory tradeoff explicitly.

Standardized major axis (SMA) regression indicated that allometric relationships between sensory structures and body size varied among species (Table 1). Eye size increased significantly with thorax width in *P. vinetorum* , *H. fasciatus* and *T. marginatus* , but not in *E. transversa* (Table 1, Fig. 1a). Funiculus size increased significantly with thorax width in *E. transversa* and *T. marginatus* , but not in *P. vinetorum* or *H. fasciatus* (Table 1, Fig. 1b). Linear models revealed significant sexual dimorphism in sensory structures for several species (Table 1, Fig. 1a-b). Eye size differed significantly between the sexes in *P. vinetorum* , *E. transversa* and *H. fasciatus* after controlling for thorax width, whereas no significant difference was detected in *T. marginatus* . Funiculus size differed significantly between the sexes in *P. vinetorum* and *E. transversa* , but not in *H. fasciatus* or *T. marginatus* . The direction of sexual dimorphism varied among species and sensory structures. Linear models provided no evidence for a sensory tradeoff between eye and funiculus size in any of the four species after controlling for thorax width and sex (all tradeoff p-values > 0.08; Table 1, Fig. 1c). Although *T. marginatus* exhibited a significant interaction between funiculus size and sex (Type II ANOVA: p-value < 0.001), the overall relationship between eye and funiculus size was not significant. This indicates that the association between sensory structures differed between males and females rather than supporting an overall sensory tradeoff.

In our study we found clear evidence of sexual dimorphism in sensory structures across hover fly species, but found no support for the sensory tradeoff hypothesis. While males and females consistently differed in their relative investment in eyes and antennae, we did not detect a general negative relationship between these traits within species. Sexual dimorphism of sensory structures was evident in three of our four species. Males generally exhibited larger eyes and larger antennae after controlling for body size and species. These patterns are likely influenced by sex-specific ecological and behavioral demands. Male Dipteran flies often have unique or enhanced visual structures such as enlarged ommatidia (Collin 1961), extra dorsal eyes (Zeil 1983) or larger eye mass (Gonzalez-Bellido et al. 2011). Previous hypotheses suggest these adaptations are driven by selection pressures such as faster mate acquisition or choice of more ornamented females (Thornhill & Alcock 1983). The former hypothesis is likely in hover flies given the propensity for males to guard air territory when searching for mates (Udayakumar et al. 2026). Our male *E. transversa* flies also exhibited larger funiculi which runs contrary to typical findings in the literature (e.g., house flies, Smallegange et al. 2008; bot flies, Zhang et al. 2012). This may demonstrate that *E. transversa* male hover flies invest in both sensory systems more than females or could potentially be the result of our methodological limitations. For example, while thorax width is commonly used as a proxy for body size in insects, this method does not take into account abdominal size differences between male and female hover flies (e.g., the ovaries and eggs in females). Despite these clear differences between males and females, we found no evidence for a sensory tradeoff in our species. The absence of a consistent negative relationship between eye and funiculus size suggests that these traits are not tightly constrained by a zero-sum allocation of resources at least at the external sensory structure level. One partial explanation in our study is the strong allometry between body size and eye and/or funiculus size in our hover flies (Table 1). This general trend for individuals with larger bodies to also have larger sensory structures may obscure or outweigh any evolutionary tradeoffs between sensory modalities. This interpretation is consistent with a recent reanalysis of dipteran sensory evolution by Farnworth & Montgomery 2022, which found that conserved allometric scaling relationships better explained variation in sensory investment than universal tradeoffs between visual and olfactory structures. Additionally, sensory tradeoffs can manifest at finer biological scales, including sensory cell investment (Sukontason et al. 2008, Singh & Mohan 2013, Keesey et al. 2019) or neural investment in brain regions associated with visual and olfactory processing (Keesey et al. 2019, Özer & Carle 2020). Future studies incorporating three-dimensional imaging approaches, scanning electron microscopy or confocal microscopy may provide a more comprehensive understanding of sensory investment in hover flies. Although we found no evidence for a general sensory tradeoff, *T. marginatus* exhibited a species-specific pattern in which the relationship between eye and funiculus size differed between males and females. This pattern may reflect sex-specific selection on sensory investment, potentially arising from differences in mating or foraging behavior between the sexes. Alternatively, species-specific ecological pressures, such as habitat use or reliance on visual versus olfactory cues, may contribute to differences in the scaling of sensory structures among these hover flies. *T. marginatus* was the only species in our study in the subfamily Syrphinae and their unique larval life may play a role in their sensory evolution. Importantly, the positive rather than the negative interaction in *T. marginatus* does not indicate a sensory tradeoff, but instead suggests that the relationship between visual and olfactory investment may vary between the sexes. Further comparative studies incorporating behavioral and ecological data across hover fly species could help determine whether these species-specific patterns reflect differences in sensory ecology or other aspects of their evolutionary history. Together, our findings suggest that while sensory systems are subject to evolutionary pressures, they are not necessarily governed by tradeoffs in all contexts. Instead, our results support the growing view that conserved allometric scaling and lineage-specific selective pressures may better explain patterns of sensory investment than universal developmental tradeoffs between sensory modalities (Farnworth & Montgomery 2022). Future studies should further investigate sensory tradeoffs at multiple biological scales to provide insight into the nuances of how evolutionary pressures shape sensory systems in animals.

## Methods

*Flies*

We selected four hover fly species ( *Palpada vinetorum* , *Eristalis transversa* , *Helophilus fasciatus* and *Toxomerus marginatus* ) that are abundant in the American midwest. Flies were netted at three sites in the St. Louis, MO area: EarthDance Organic Farm, University of Missouri – St. Louis south campus prairie and the Litzsinger Road Ecology Center. We euthanized via freezing and pinned the flies for microscopy photography.

*Morphometric Measurements*

We placed each individual fly and a .5 mm ruler under a Leica M80 dissecting microscope with a camera attachment (Canon EOS Rebel T3i). We took one dorsal and one frontal photograph of each fly. We then used these to measure thorax width, eye width/length and funiculus width/length using ImageJ 1.54d. Thorax width was used as a proxy for total body size. The funiculus is the third antennal segment and contains the highest density of sensilla (olfactory hairs). Thus, we decided to use funiculus size as a proxy for antennal size following Keesey et al. 2019. Average eye and funiculus size for each fly was calculated from these morphometric measurements.

*Analyses*

To examine allometric relationships between sensory structures and body size, we used standardized major axis (SMA) regression implemented in the R package smatr. Because allometric relationships are multiplicative, eye size, funiculus size and thorax width were natural log-transformed prior to analysis. We conducted separate SMA analyses for each species to examine relationships between thorax width and eye size as well as thorax width and funiculus size. We also used SMA regression to compare these relationships across species. To evaluate sexual dimorphism, we used linear models with natural log-transformed eye size or funiculus size as the response variable and natural log-transformed thorax width and sex as predictors. We used pairwise comparisons of estimated marginal means to evaluate sex differences in sensory structure size. To test for sensory tradeoffs, we used linear models with natural log-transformed eye size as the response variable and natural log-transformed funiculus size, sex and natural log-transformed thorax-width as predictors. Predictor effects in tradeoff models were evaluated using Type II ANOVA. 
